# Aromatic “Redox Tag”-assisted Diels–Alder reactions by electrocatalysis†
†Electronic supplementary information (ESI) available: Additional figures, tables and general, experimental, theoretical and spectral information. See DOI: 10.1039/c6sc02117d



**DOI:** 10.1039/c6sc02117d

**Published:** 2016-06-30

**Authors:** Yohei Okada, Yusuke Yamaguchi, Atsushi Ozaki, Kazuhiro Chiba

**Affiliations:** a Department of Applied Biological Science , Tokyo University of Agriculture and Technology , 3-5-8 Saiwai-cho, Fuchu , Tokyo 183-8509 , Japan . Email: chiba@cc.tuat.ac.jp; b Department of Chemical Engineering , Tokyo University of Agriculture and Technology , 2-24-16 Naka-cho, Koganei , Tokyo 184-8588 , Japan

## Abstract

Electrocatalysis was employed to promote Diels–Alder reactions between electronically mismatched substrates. A catalytic amount of electricity was enough to complete the overall reactions and GC-MS monitoring and CV measurements clearly illustrated the electrocatalytic nature of the reactions, which involve an EC-backward-E mechanism. The electrocatalytic Diels–Alder reactions were rationally designed based on the concept of redox tags. The results were supported by DFT calculations.

## Abstract 2

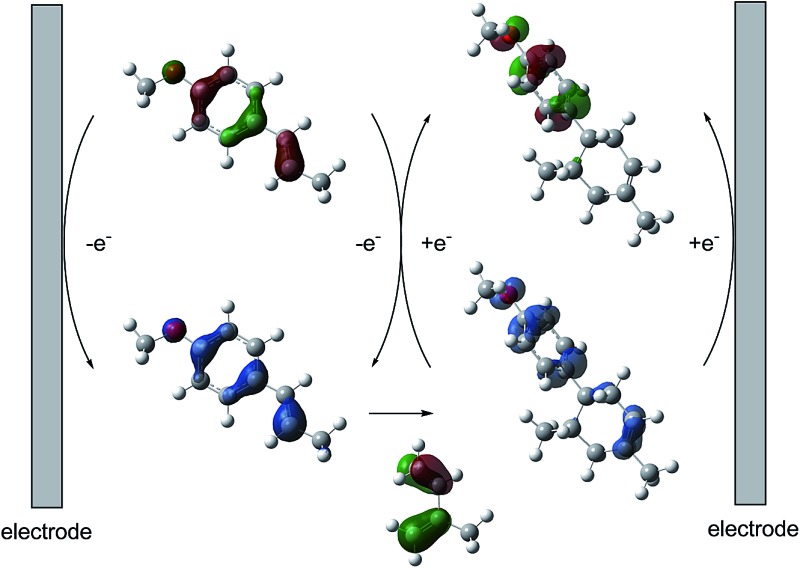
Electrocatalytic Diels–Alder reactions have been designed and demonstrated based on the aromatic “redox tag” concept.

## Introduction

Extensive effort to synthesize highly complex organic molecules with improved atom, step and redox economy has resulted in the development of several important classes of catalytic reactions. While transition metal catalysis has dominated the synthetic landscape over the past century, organo- and photocatalysis have recently been recognized as promising approaches for the activation of small molecules. Stemming from the pioneering work of List and MacMillan in 2000,^1^ organocatalysis has grown explosively, demonstrating that not only prolines and imidazolidinones, but also several other small organic molecules, could act as organocatalysts.^2^ Since MacMillan and Yoon introduced the use of a ruthenium sensitizer in 2008,^3^ photocatalysis has occupied a central place in the study of new catalytic reactions.^4^ The sensitizer can be excited by irradiation with visible light (*λ*_max_ = 452 nm) to induce one-electron transfer, while most organocatalysis is restricted to two-electron pathways. Nowadays, some catalytic methods have been elegantly merged to achieve complementary catalytic systems, promoting reactions that otherwise only occur with difficulty.

Arguably, the most simple and straightforward one-electron transfer process is the electrochemical method, which facilitates a redox reaction at the surface of an electrode.^5^ The oxidizing and reducing power can be precisely controlled in a switchable manner, providing selective electron transfer events based on the redox potential of the substrates. Although polar organic solvents and supporting electrolytes are required to achieve conductivity, processes in which the only net reagent is the electron itself are possible, resulting in an extremely environmentally friendly procedure. In particular, if a catalytic amount of electricity is enough to complete an overall transformation, in a process known as an electrocatalytic reaction, this constitutes a powerful option for the synthesis of organic molecules. Electrocatalysis has been extensively investigated in the field of inorganic chemistry, but it has not yet found substantial application in organic synthesis, possibly due to the difficulty of designing reactions. This is because electrocatalytic reactions must involve a chain mechanism in which the intermediate and/or product electrochemically generated *in situ* activates the starting material. Such reaction design must balance the redox potentials of all the reactants, intermediates and product; otherwise, undesired redox processes will take place.

The Diels–Alder reaction is a classic reaction, which is still at the forefront of the carbon–carbon bond formation toolbox, and has been the subject of modern research in synthetic chemistry. The reaction mechanism has been particularly well studied, and the scope expanded to include a wide variety of diene/dienophile combinations. The only thing that needs to be considered is electronic matching; more specifically, that one reaction partners should be electron-rich and the other electron-deficient. However, even this hurdle can be overcome based on the single-electron-transfer strategy.^6^ A typical example is the reaction between an electron-rich diene and a dienophile. Although these combinations are electronically mismatched, one-electron oxidation by chemical oxidants^7^ or photosensitizers^8^ can produce a radical cation of one component, which is then trapped by the other component.

We have been developing methods for intermolecular carbon–carbon bond formation by electrocatalysis.^9^ Our reaction design involves the aromatic “redox tag” concept^10^—that is, an aromatic ring that provides both the oxidant and the reductant in the overall electrochemical transformation. Since the aromatic ring stabilizes all radicals, ions and radical ions to a significant extent, and the redox potential can be fine-tuned based on the number and/or position of substituents, programmed electron-transfer events may be achieved. Described herein is the design and development of aromatic redox-tag-assisted Diels–Alder reactions by electrocatalysis.

## Results and discussion

**Fig. 1:**
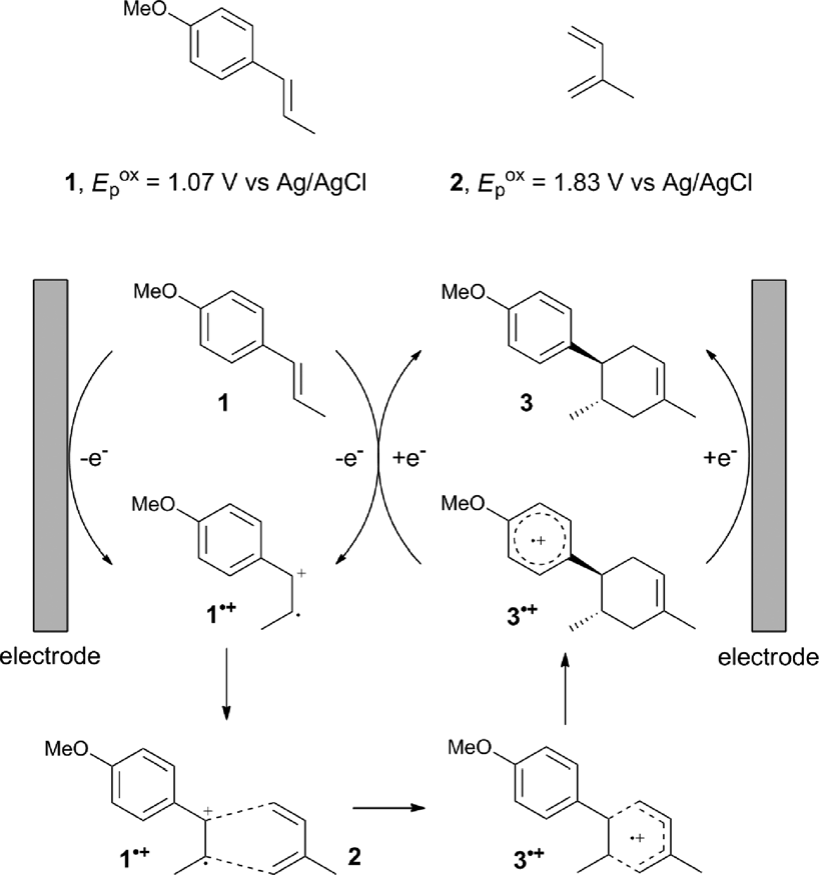
Current working model and expected mechanism at the surface of an electrode.

**Scheme 1:**
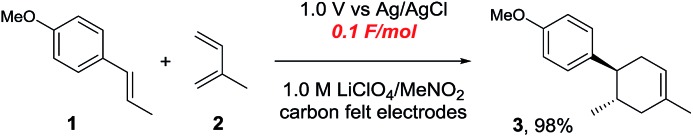
Electrocatalytic Diels–Alder reaction between *trans*-anethol (**1**) and isoprene (**2**).

**Fig. 2:**
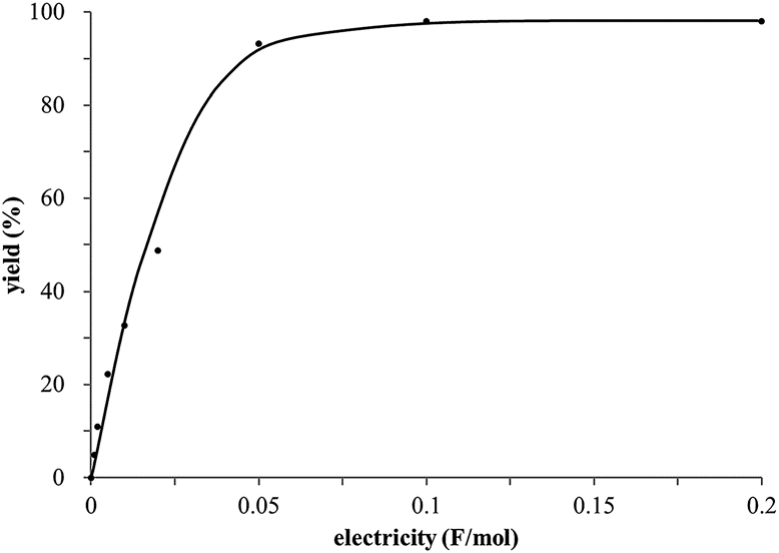
GC-MS monitoring of the electrocatalytic Diels–Alder reaction between **1** and **2**.

The study began with the reaction between *trans*-anethol (**1**) and isoprene (**2**), which has recently been demonstrated as a useful application of photocatalysis in organic synthesis,^11^ using the electrocatalytic conditions we have developed. The reaction is well-established and understood to involve a radical cation chain process.^12^ Based on the oxidation potentials, *trans*-anethol (**1**, *E*oxp = 1.07 V *vs.* Ag/AgCl) was selectively oxidized at the anode even in the presence of isoprene (**2**, *E*oxp = 1.83 V *vs.* Ag/AgCl). We speculated that the aromatic radical cation **3˙^+^** would form through intermolecular carbon–carbon bond formation between the anodically generated anethol radical cation **1˙^+^** and isoprene (**2**), with a higher oxidation potential than **1**. The aromatic radical cation **3˙^+^** could then oxidize the starting *trans*-anethol (**1**), triggering a radical cation chain mechanism and completing the overall reaction with a catalytic amount of electricity, while the reduction of **3˙^+^** at the electrode would increase the total electrical input (Fig. 1). We questioned whether the aromatic ring of **1** might function as a redox tag to achieve an electrocatalytic Diels–Alder reaction. Therefore, we attempted anodic oxidation of **1** in the presence of 2 equiv. of **2** at 1.00 V *vs.* Ag/AgCl. The results were better than expected: the reaction was completed with only 0.1 F mol^–1^ of electricity to give the Diels–Alder adduct **3** in excellent yield, suggesting that the aromatic radical cation **3˙^+^** was reduced preferentially by **1** rather than at the electrodes (Scheme 1). The electrocatalysis was demonstrated by GC-MS measurements (Fig. 2). The monitoring curve clearly illustrated the electrocatalytic nature of the reaction, and the reaction was almost completed with only 0.05 F mol^–1^ of electricity.

The control studies and optimization of the conditions for the electrocatalytic Diels–Alder reaction between **1** and **2** are summarized in Table 1. The reaction did not take place without electricity (entry 2), suggesting that the substrate combination was indeed electronically mismatched. A lower concentration of LiClO_4_ gave slightly lower yields of the Diels–Alder adduct **3** (entries 3 and 4), while NaClO_4_ and Et_4_NClO_4_ were much less effective (entries 5 and 6). LiClO_4_ could not be replaced by either LiBF_4_ or LiPF_6_ (entries 7 and 8), suggesting that both Li^+^ and ClO_4_^–^ were crucial for the reaction. When MeCN or MeOH was used instead of MeNO_2_, once again the yield dropped off (entries 9 and 10), which showed that the combination of LiClO_4_ and MeNO_2_ was the best electrolyte solution for the reaction.

**Table 1:** Control studies and optimization of conditions for electrocatalytic Diels–Alder reaction between **1** and **2** ^*a*^

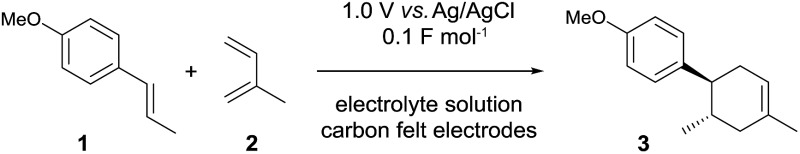			
Entry	Supporting electrolyte	Solvent	Yield^*b*^ (%)
1	1.0 M LiClO_4_	MeNO_2_	98
2^*c*^	1.0 M LiClO_4_	MeNO_2_	0
3	0.2 M LiClO_4_	MeNO_2_	80
4	0.1 M LiClO_4_	MeNO_2_	72
5	1.0 M NaClO_4_	MeNO_2_	33
6	1.0 M Et_4_NClO_4_	MeNO_2_	3
7	1.0 M LiBF_4_	MeNO_2_	30
8	1.0 M LiPF_6_	MeNO_2_	0
9	1.0 M LiClO_4_	MeCN	26
10	1.0 M LiClO_4_	MeOH	0

^*a*^All reactions were carried out on a 1.60 mmol scale of anethol (**1**) with 2 equiv. of isoprene (**2**) in 20 mL of electrolyte solution at room temperature.

^*b*^Yields were determined by NMR using benzaldehyde as an internal standard.

^*c*^No electricity was applied.

**Fig. 3:**
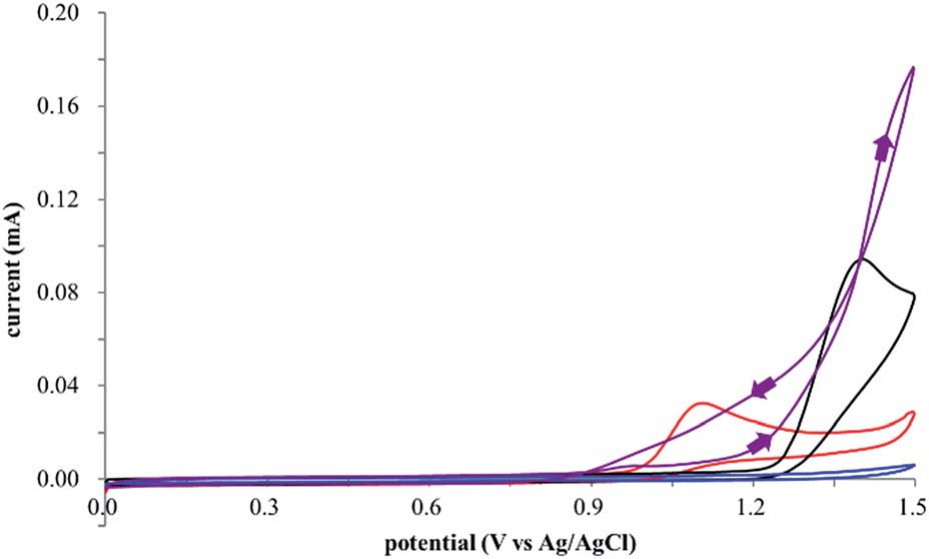
Cyclic voltammograms of 2 mM **1** (red line), 160 mM **2** (blue line), 2 mM of the Diels–Alder adduct **3** (black line), and 2 mM **1** in the presence of 160 mM **2** (purple line).

**Fig. 4:**
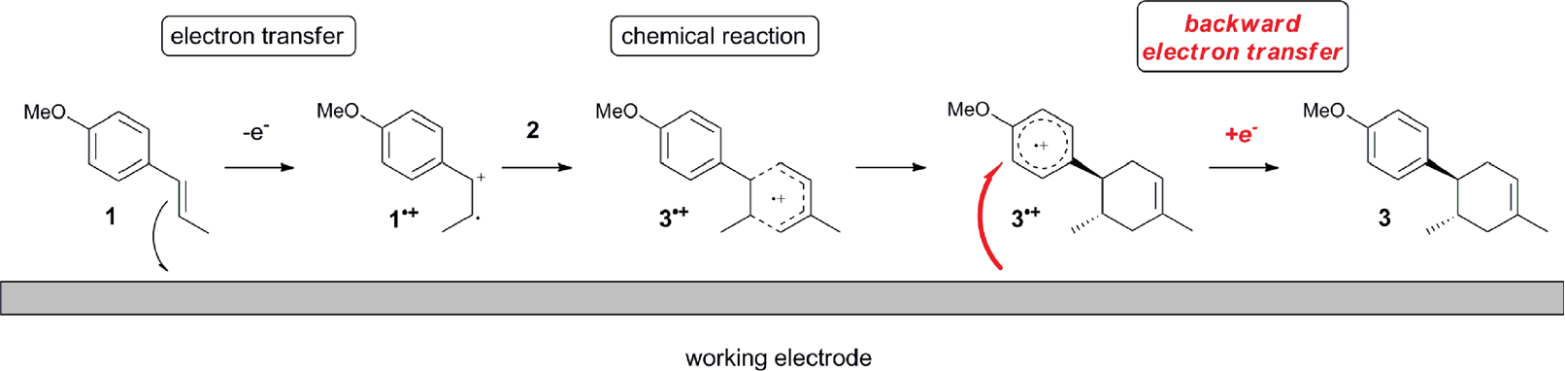
Plausible EC-b-E mechanism at the surface of the working electrode.

The electrocatalysis was further investigated by cyclic voltammetry (CV) measurements (Fig. 3). The potential was swept from 0 to 1.50 V *vs.* Ag/AgCl at a rate of 0.50 V s^–1^, and typical irreversible redox waves were observed for **1** and the Diels–Alder adduct **3**, including clear oxidation peaks at 1.07 and 1.40 V *vs.* Ag/AgCl, respectively. As expected, the oxidation peak of isoprene (**2**, *E*oxp = 1.83 V *vs.* Ag/AgCl) was not seen in this sweep range. As hypothesized, the oxidation potential of **3** was significantly greater than that of **1**; thus, it was reasonable to assume that the aromatic radical cation **3˙^+^** efficiently oxidized **1** to induce the electrocatalytic Diels–Alder reaction through a radical cation chain mechanism. Moreover, when the potential was swept for **1** in the presence of **2**, the oxidation wave of **1** was not observed to a significant extent, and that of the product (**3**) was not saturated at 1.40 V *vs.* Ag/AgCl, demonstrating a much greater current. This wave could be unambiguously explained as being due to an EC-backward-E (E: electron transfer, C: chemical reaction) mechanism (Fig. 4).^13^ Thus, **1** is initially oxidized by the working electrode at around 1.00 V *vs.* Ag/AgCl and higher potentials to generate the corresponding radical cation **1˙^+^** (E), which is immediately trapped by **2**, affording the aromatic radical cation **3˙^+^** (C). As the oxidation potential of the product (**3**) is 1.40 V *vs.* Ag/AgCl, the aromatic radical cation **3˙^+^** is reduced by the working electrode to form the neutral product **3** (backward E), decreasing the overall current. At around 1.20 V *vs.* Ag/AgCl and higher potentials, **3** is then oxidized by the working electrode, showing the oxidation wave. During the sweep, **1** was continuously oxidized to the corresponding radical cation **1˙^+^**, triggering the reaction. It should be noted that the aromatic radical cation **3˙^+^** could also oxidize **1** to induce the reaction, resulting in accumulation of the Diels–Alder adduct (**3**), which results in a greater current even under a reducing sweep direction. In bulk electrolysis, both the EC-backward-E and radical cation chain mechanisms are possible, which account for the electrocatalytic nature, while the EC-backward-E mechanism alone is much more likely to explain the decrease of the oxidation wave of **1** in the CV

Based on this mechanistic understanding, we then focused on the scope of the electrocatalytic Diels–Alder reaction (Table 2; see Fig. S1–S7† for cyclic voltammograms). First, several simple dienes (**4–8**) were tested in place of **2** to study the conformational effect on the reaction. As expected, the Diels–Alder adduct **10** was obtained in excellent yield from 2,3-dimethylbuta-1,3-diene (**5**) with no difficulty (entry 3). The Diels–Alder adduct **11** was also achieved in excellent yield from (*E*)-2-methylpenta-1,3-diene (**6**) (entry 4), while the use of (*Z*)-2-methylpenta-1,3-diene (**7**) gave no Diels–Alder adduct (entry 5). Butadiene (**4**) and 2,4-dimethylpenta-1,3-diene (**8**) were less effective for the reaction, giving the Diels–Alder adducts (**9**, **13**) in low to good yield (entries 1 and 6). This can be explained based on the much-studied Diels–Alder theory, which states that the cisoid forms of the dienes are required.

**Table 2:** Range of dienes for electrocatalytic Diels–Alder reactions of **1** ^*a*^

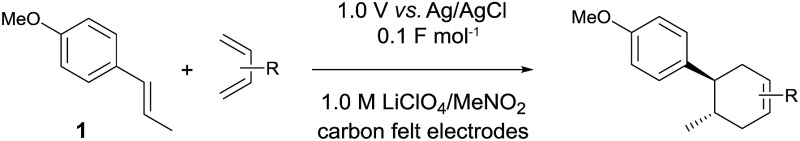		
Entry	Diene	Diels–Alder adduct, yield^*b*^
1	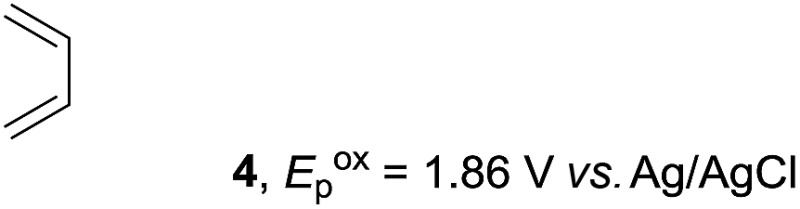	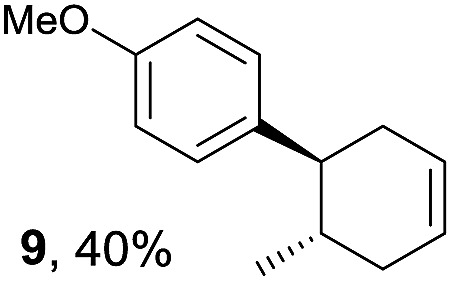
2	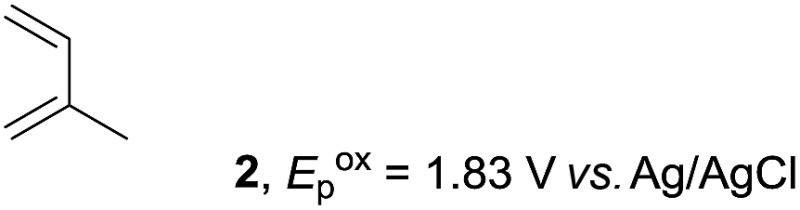	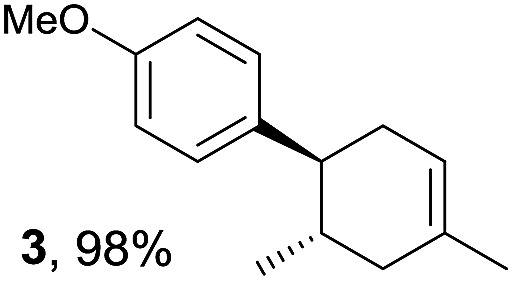
3	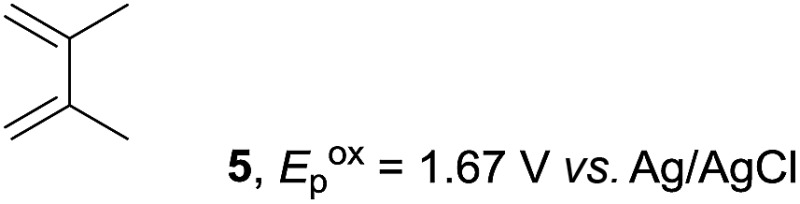	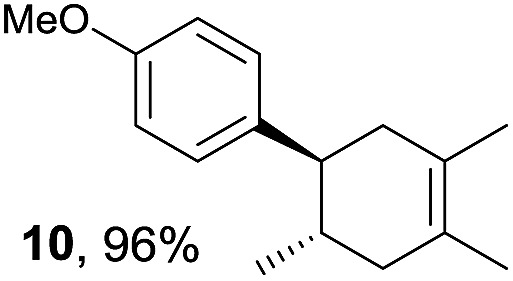
4	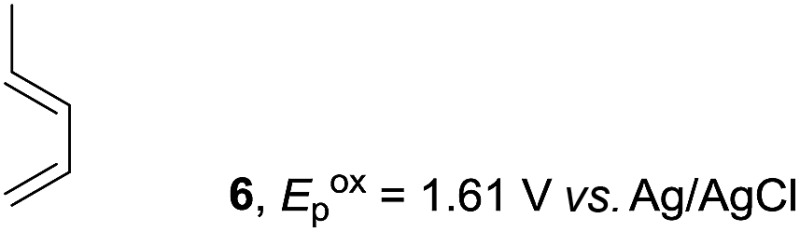	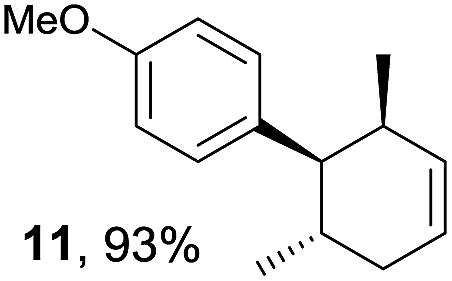
5	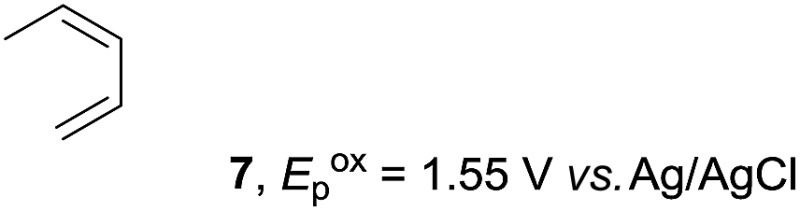	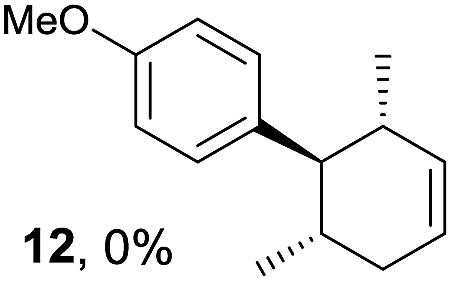
6	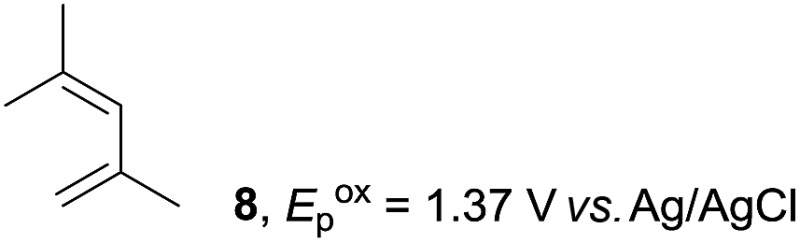	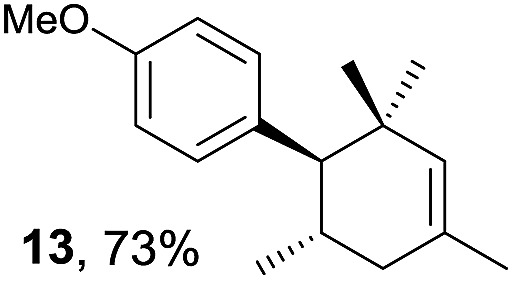

^*a*^All reactions were carried out in 1.60 mmol scale of *trans*-anethol (**1**) with 2 equiv. of diene (**2**, **4–8**) in 20 mL of electrolyte solution at room temperature.

^*b*^Yields were determined by NMR using benzaldehyde as an internal standard.

Despite the reasonable range of dienes, the number of styrene structures that were successful in the reaction was disappointingly low (Table 3; see Fig. S8–S14† for cyclic voltammograms). As found in an earlier report by Yoon, *trans*-β-methylstyrene (**14**) was not effective (entry 1). In other words, an unsubstituted phenyl ring is unable to function as a redox tag. Although we previously found that a mesityl group acted as a redox tag in electrocatalytic [2 + 2] cycloadditions,^10*a*^ the Diels–Alder adduct **22** was only obtained in low yield from styrene **15** (entry 2). When a methoxy group was introduced onto the *ortho*-position of the aromatic ring, the oxidation potential was significantly greater than that of **1** and the reaction yield decreased significantly (entry 3). Presumably, *ortho*-substitution can also cause steric effects, thereby inhibiting the reaction. It should be noted that the reaction of *cis*-anethol (**17**) selectively gave the *trans*-Diels–Alder adduct **3**, suggesting that the stereochemistry of the styrene is not retained throughout the reaction, probably because the styrene double bonds can rotate while in the radical cation form (entry 4). Further installation of methoxy groups onto the *ortho*- and/or *meta*-position of the aromatic rings also significantly decreased the reaction yield (entries 6–8). This can be explained by the fact that styrenes **18–20** are highly reactive in the electrolyte solution leading to polymerization. Indeed, insoluble solids were formed in the presence of high concentrations of LiClO_4_; for this reason, a low concentration must be used for styrenes **18–20**.

**Table 3:** Range of styrenes for electrocatalytic Diels–Alder reactions of **2** ^*a*^

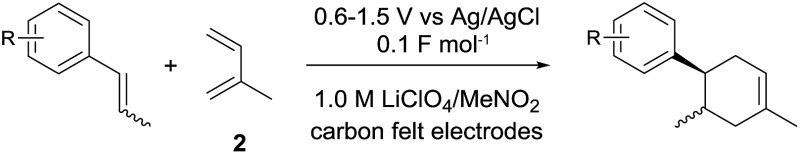		
Entry	Styrene	Diels–Alder adduct, yield^*b*^
1	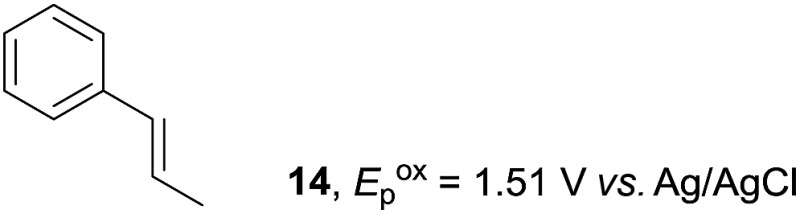	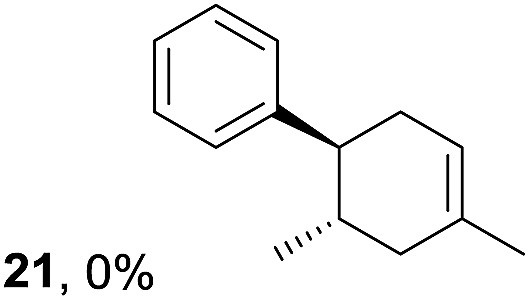
2	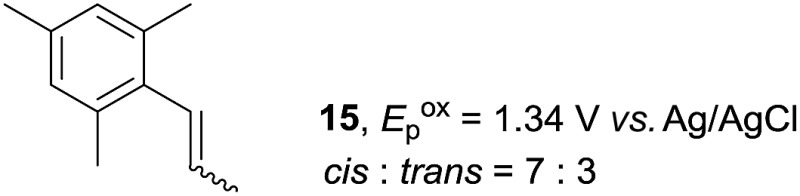	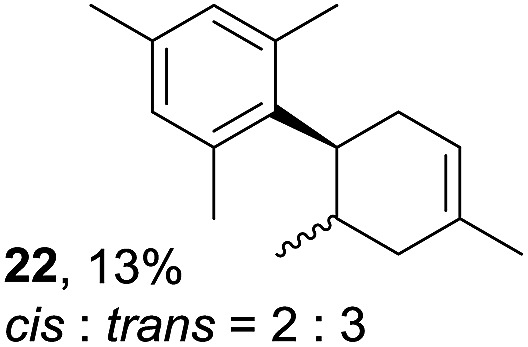
3	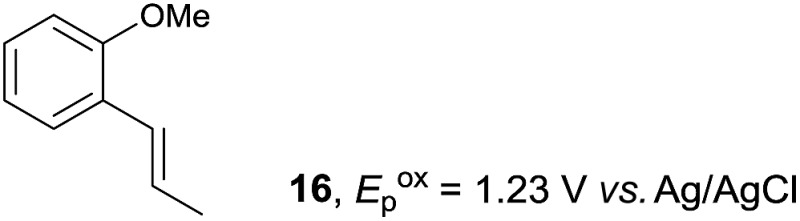	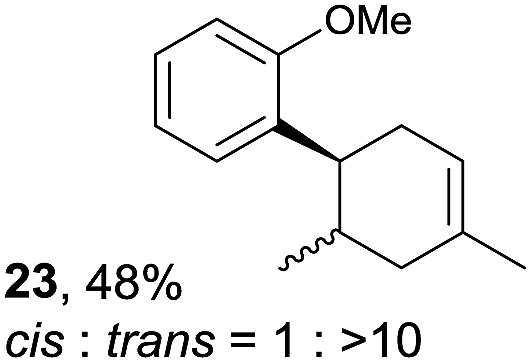
4	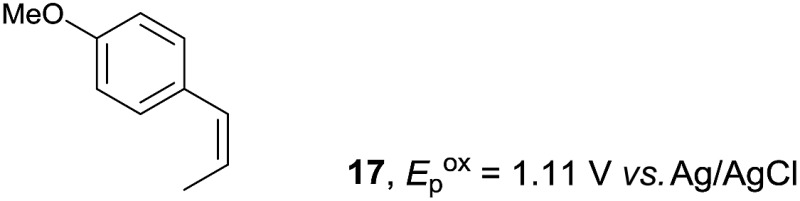	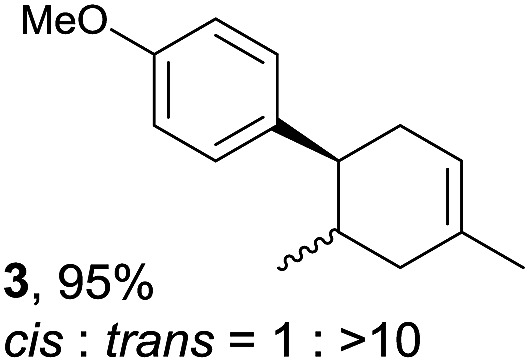
5	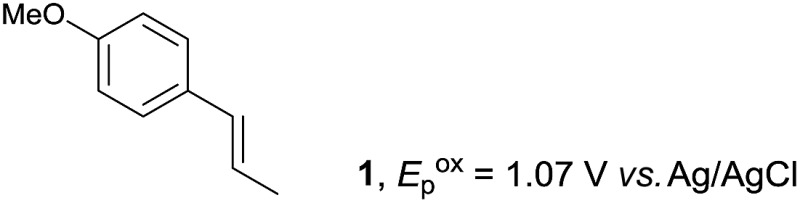	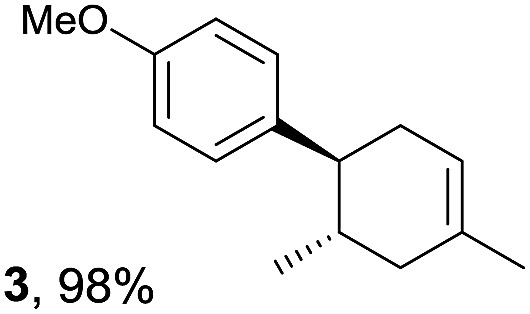
6^*c*^	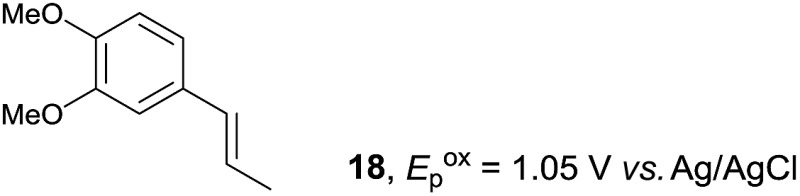	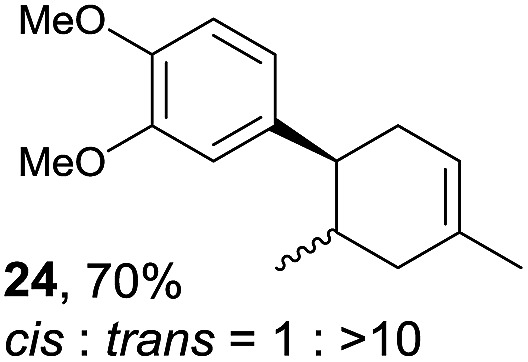
7^*c*^	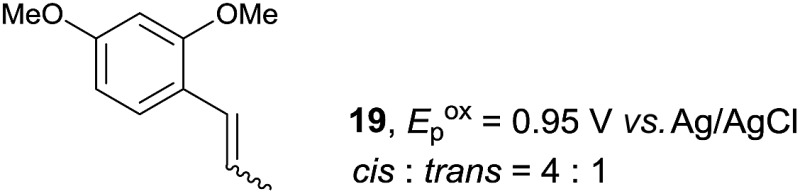	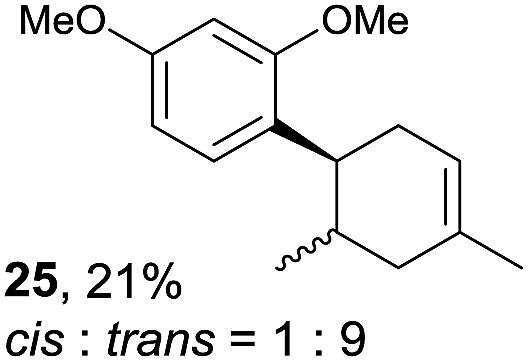
8^*c*^	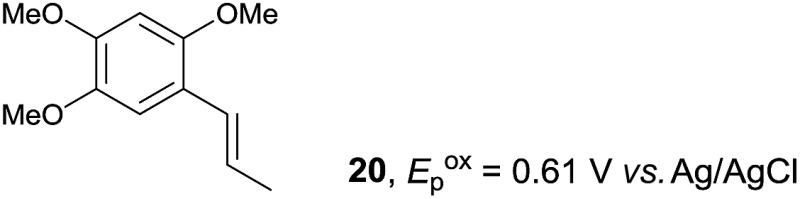	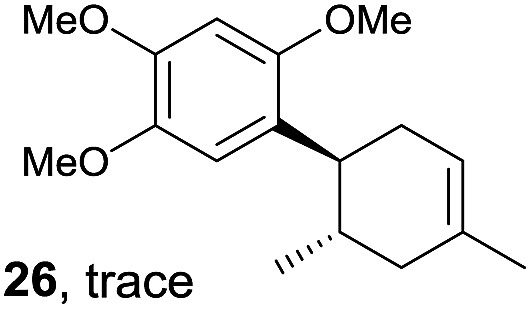

^*a*^All reactions were carried out using a 1.60 mmol scale of styrene (**1**, **14–20**) with 2 equiv. of isoprene (**2**) in 20 mL of electrolyte solution at room temperature.

^*b*^Yields were determined by NMR using benzaldehyde as an intenal standard.

^*c*^0.5 F mol^–1^ was applied in 0.20 M LiClO_4_/MeNO_2_.

Normal Diels–Alder reactions are thought to take place in a concerted manner, involving two simultaneous bond formations, so that the stereochemistries of the diene and the dienophile are fully retained. However, electrocatalytic Diels–Alder reactions can also take place in a stepwise fashion, since there are at least two steps, including electron transfer and bond formation. Indeed, the stereochemistry of the styrenes is not reflected in the Diels–Alder adducts, which implies that bond rotation is possible.

**Fig. 5:**
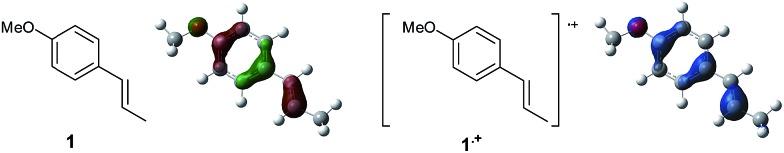
HOMO (isovalue = 0.06) of **1** (left) and spin density (isovalue = 0.004) of the corresponding radical cation **1˙^+^** (right).

**Fig. 6:**
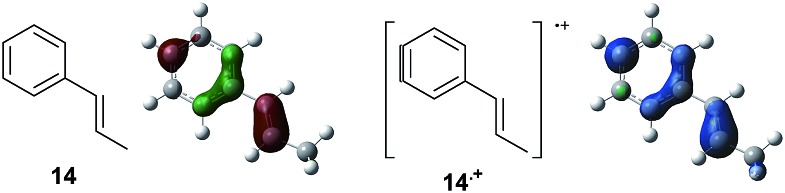
HOMO (isovalue = 0.06) of **14** (left) and spin density (isovalue = 0.004) of the corresponding radical cation **14˙^+^** (right).

To gain further insight into the mechanism, density functional theory (DFT) calculations were carried out at the B3LYP/6-311G(2d,2p) level for the reaction of **1** and *trans*-β-methylstyrene (**14**) with **2**. Although **14** did not give the Diels–Alder adduct **21**, it was expected to be a rational model for a theoretical approach. Since the applied potential at the surface of the electrodes can be controlled in a switchable manner - indeed, a higher value of 1.50 V *vs.* Ag/AgCl was used for the reaction of **14** - the corresponding radical cation **14˙^+^** could still be generated. The optimized structures of **1** and **14** show that the HOMOs are similarly located at the aromatic ring and the double bond (Fig. 5 and 6; see Tables S1–S4† for Cartesian coordinates).

The HOMOs were expected to be used for anodic oxidation to generate the corresponding radical cations, which would be characterized by Mulliken positive charges and spin densities. To our surprise, no significant Mulliken positive charges were observed on the double bonds of the radical cations **1˙^+^** and **14˙^+^**, while the spins were distributed along the HOMOs of the neutral forms (see Fig. S15† for Mulliken positive charges). In particular, the spin densities of the β-positions were greater than those of the α-positions, indicating that trapping by **2** would occur at the β-position in homolytic fashion.

**Fig. 7:**
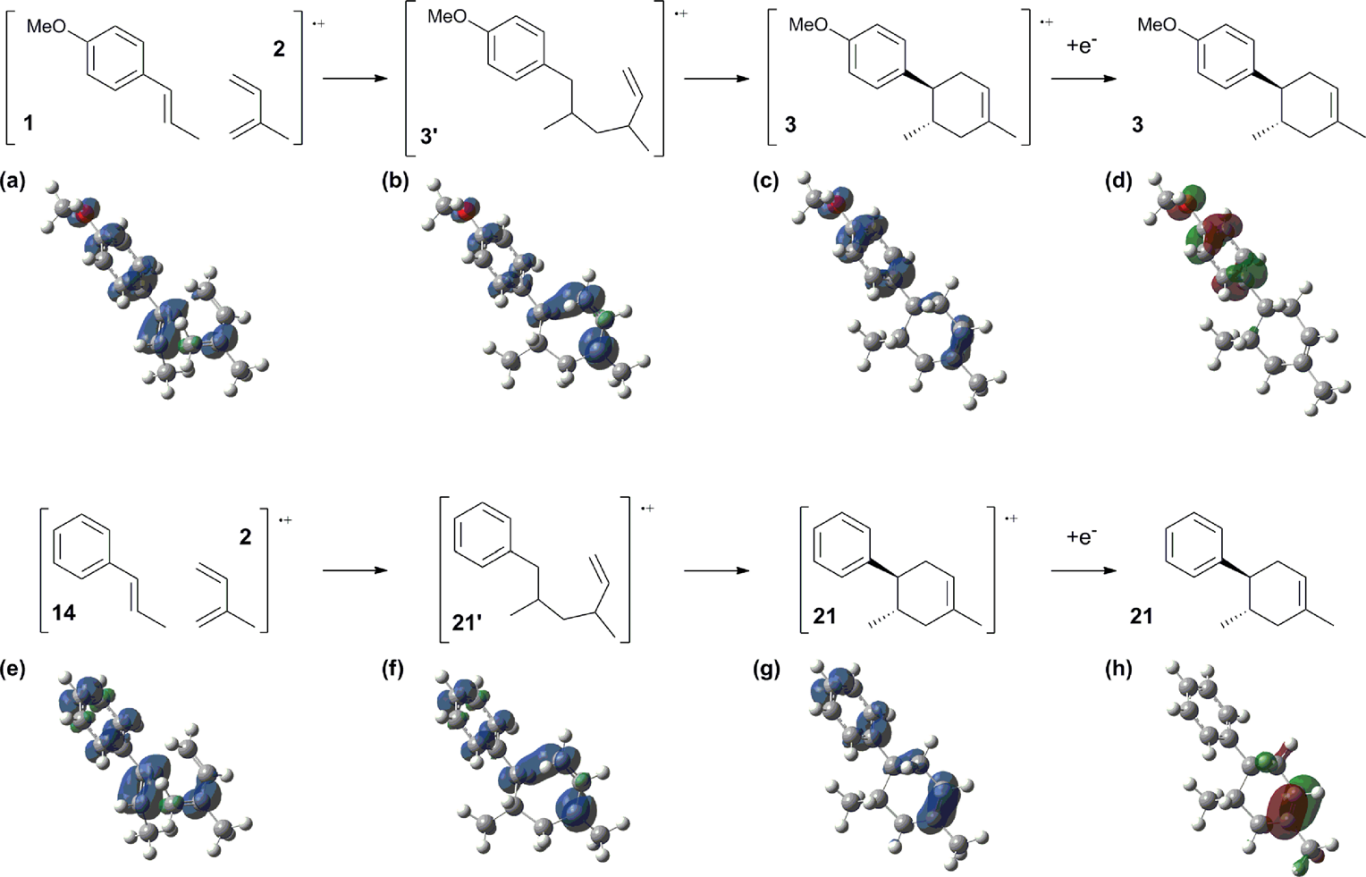
Spin densities (isovalue = 0.004) of the plausible reaction intermediates (a–c and e–g) and HOMOs (isovalue = 0.06) of **3** (d) and **21** (h).

Based on this hypothesis, the stepwise bond-forming mechanism was then used for subsequent calculations (Fig. 7; see Tables S1–S4† for Cartesian coordinates). When the distances between **1** or **14** and **2** are far enough to prevent bond formation, the spins are mainly distributed on **1** and **14**, suggesting that selective oxidation of **1** or **14** is possible even in the presence of **2** (a and e). On the other hand, no spins are localized on the β-positions after the initial bond formations, indicating that trapping by **2** would proceed in homolytic fashion (b and f). During the second bond formation, significant spins are shifted from the cyclohexene moieties to the aromatic rings, which could be explained as intramolecular electron transfer (c and g). While the spin densities of the aromatic ring are greater than those of the cyclohexene moiety for the reaction intermediate formed between **1** and **2** (c), this is not the case for the reaction intermediate formed between **14** and **2** (g). These observations indicate that intramolecular electron transfer from the methoxyphenyl ring to the cyclohexene moiety is more efficient than that from the non-substituted phenyl ring to the cyclohexene moiety. Moreover, while the HOMOs of **1** and **14** are similarly located over the molecules, the corresponding Diels–Alder adducts **3** and **21** show unique HOMOs. In **3**, the HOMO is dominantly localized on the methoxyphenyl ring, suggesting that oxidation - and thus reduction of the corresponding radical cation **3˙^+^** - takes place on the methoxyphenyl ring (d), and that the methoxyphenyl ring reduces the cyclohexene radical cation to construct the neutral form. Meanwhile, for **21**, the HOMO is dominantly localized on the cyclohexene moiety, suggesting that oxidation - and thus reduction of the corresponding radical cation **21˙^+^** - takes place on the cyclohexene moiety (g), which means that the non-substituted phenyl ring is unable to reduce the cyclohexene radical cation to construct the neutral form.

## Conclusions

In conclusion, we successfully designed and demonstrated electrocatalytic Diels–Alder reactions based on the aromatic redox tag concept. The electrocatalytic nature of the reaction was demonstrated by GC-MS monitoring and CV measurements, with the reaction clearly exhibiting an EC-backward-E mechanism. The function of the aromatic redox tag was also supported by DFT calculations, indicating that intramolecular electron transfer from the aromatic ring to the cyclohexene moiety is key. We believe that the experimental and computational results described herein are advantageous in furthering the design and development of reactions directed by electrocatalysis.
