# Supplementary material for: Aromatic “Redox Tag”-assisted Diels–Alder reactions by electrocatalysis
Source: Chem Sci. 2016 Jun 30;7(10):6387–93. doi: 10.1039/c6sc02117d (PMC5363793; doi:10.1039/c6sc02117d)
Supplement: Supplementary file 1 [file SC-007-C6SC02117D-s001.docx]

Supporting Information

**Aromatic “Redox Tag” Assisted Diels-Alder Reactions by Electrocatalysis**

**Yohei Okada,^b^ Yusuke Yamaguchi,^a^ Atsushi Ozaki,^a^ Kazuhiro Chiba*^a^**

^a^Department of Applied Biological Chemistry, Tokyo University of Agriculture and Technology, 3-5-8 Saiwai-cho, Fuchu, Tokyo 183-8509, Japan

^b^Department of Chemical Engineering, Tokyo University of Agriculture and Technology, 2-24-16 Naka-cho, Koganei, Tokyo 184-8588, Japan

Tel: (+81)-42-367-5667

Fax: (+81)-42-367-5667

E-mail: chiba@cc.tuat.ac.jp

Table of contents:

1. Additional Figures and Tables

2. General Information

3. Experimental and Spectra Information of Starting Materials

4. Experimental and Spectra Information of Diels-Alder Adducts

5. Theoretical Information

6. Copies of NMR Spectra

**1. Additional Figures and Tables**

**Figure S1.** Cyclic Voltammogram of *trans*-Anethole **1**.

**Figure S2.** Cyclic Voltammogram of Isoprene **2**.

**Figure S3.** Cyclic Voltammogram of Buta-1,3-diene (**4**).

**Figure S4.** Cyclic Voltammogram of 2,3-Dimethylbuta-1,3-diene (**5**).

**Figure S5.** Cyclic Voltammogram of *trans*-Penta-1,3-diene (**6**).

**Figure S6.** Cyclic Voltammogram of *cis*-Penta-1,3-diene (**7**).

**Figure S7.** Cyclic Voltammogram of 2,4-Dimethylpenta-1,3-diene (**8**).

**Figure S8.** Cyclic Voltammogram of *trans*-β-Methylstyrene **14**.

**Figure S9.** Cyclic Voltammogram of β-Methyl-2,4,6-trimethylstyrene **15**.

**Figure S10.** Cyclic Voltammogram of β-Methyl-2-methoxystyrene **16**.

**Figure S11.** Cyclic Voltammogram of *cis*-Anethole **17**.

**Figure S12.** Cyclic Voltammogram of *trans*-β-Methyl-3,4-dimethoxystyrene **18**.

**Figure S13.** Cyclic Voltammogram of β-Methyl-2,4-dimethoxystyrene **19**.

**Figure S14.** Cyclic Voltammogram of *trans*-β-Methyl-2,4,5-trimethoxystyrene **20**.


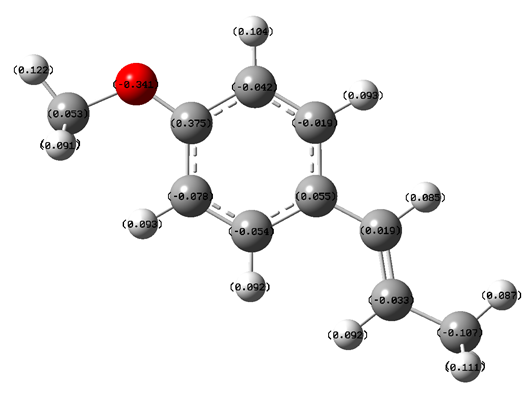


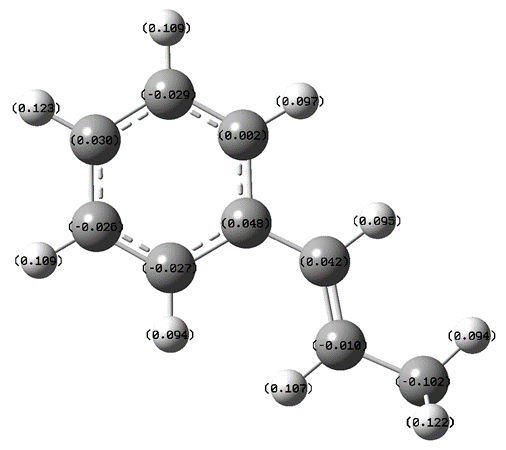


**Figure S15.** Mulliken Positive Charges of the Radical Cation **1^.+^** (Above) and the Radical Cation **14^.+^** (Below).


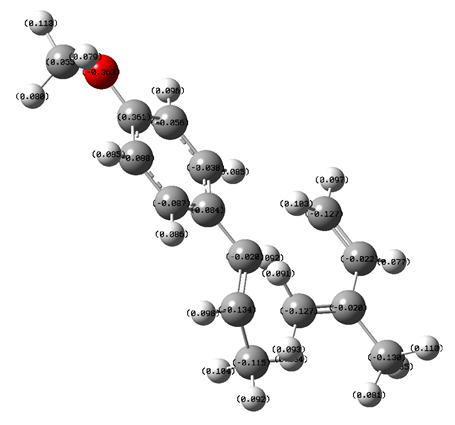

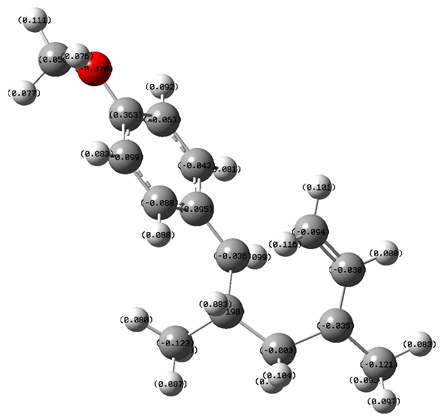


**Figure S16.** Mulliken Positive Charges of the Reaction Intermediates Figure7-a (Above) and Figure 7-b (Below).


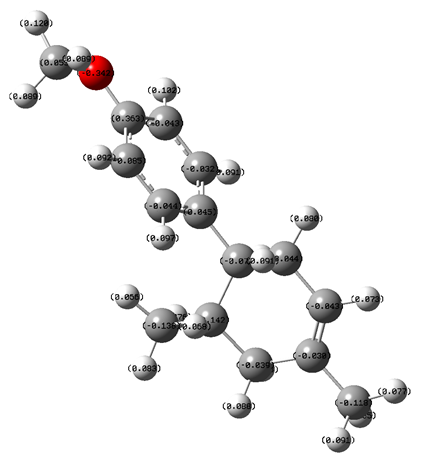


**Figure S17.** Mulliken Positive Charges of the Radical Cation **3^.+^**, Figure 7-c.


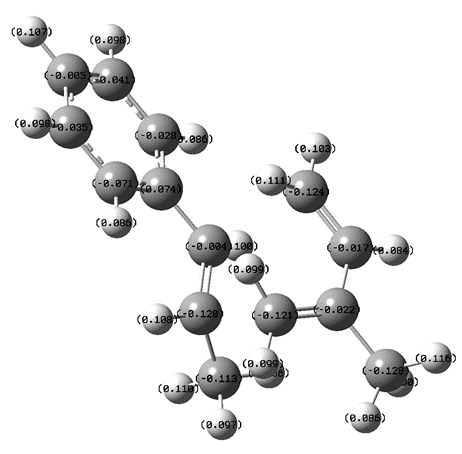


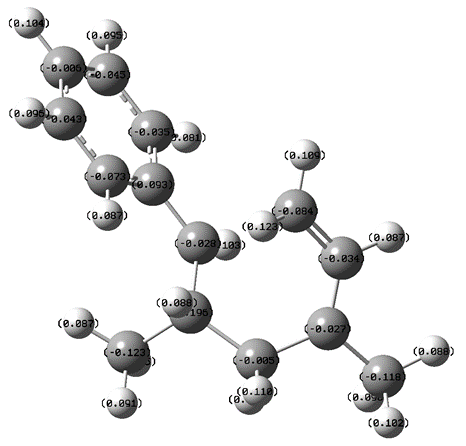


**Figure S18.** Mulliken Positive Charges of the Reaction Intermediates Figure7-e (Above) and Figure 7-f (Below).

**
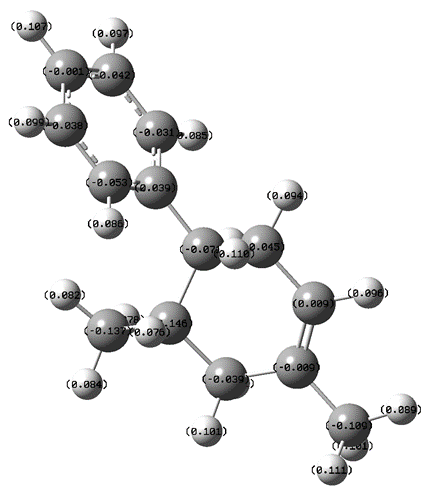
**

**Figure S19.** Mulliken Positive Charges of the Radical Cation **21^.+^**, Figure 7-g.

**Table S1. Cartesian Coordinates of the Optimized Structure for the *trans*-Anethole 1 Calculated at the RB3LYP/6-311G(2d,2p) level.**

Atom X Y Z

C 0.2123670 -0.9016734 -0.0000701

C 0.9111684 0.3160533 0.0001112

C 0.1408795 1.4956616 0.0001044

C -1.2464180 1.4657700 -0.0000448

C -1.9216027 0.2379193 -0.0002311

C -1.1805526 -0.9515205 -0.0002668

H 0.7603866 -1.8396769 -0.0000536

H 0.6485587 2.4577069 0.0002562

H -1.8307613 2.3807284 -0.0000101

H -1.6754130 -1.9162448 -0.0004902

O -3.2858048 0.3059632 -0.0005713

C -4.0181385 -0.9082542 0.0005796

H -5.0718161 -0.6228186 0.0012972

H -3.8067332 -1.5093579 -0.8939386

H -3.8052561 -1.5085478 0.8952803

C 2.3773893 0.4192139 0.0002427

H 2.7576070 1.4423664 0.0006044

C 3.2758720 -0.5771749 -0.0001326

H 2.9363821 -1.6132866 -0.0005713

C 4.7634361 -0.3807120 0.0000351

H 5.2289502 -0.8465440 0.8799685

H 5.2290554 -0.8457555 -0.8802616

H 5.0290757 0.6820262 0.0005243

**Table S2. Cartesian Coordinates of the Optimized Structure for the Radical Cation of the *trans*-Anethole 1^.+^ Calculated at the UB3LYP/6-311G(2d,2p) level.**

Atom X Y Z

C -0.2224841 -0.9201597 0.0000660

C -0.9174481 0.3341168 0.0000216

C -0.1316348 1.5321326 -0.0000522

C 1.2352980 1.4878473 -0.0000786

C 1.9029910 0.2264806 -0.0000505

C 1.1476610 -0.9816370 0.0000305

H -0.7840307 -1.8477273 0.0001377

H -0.6403421 2.4914864 -0.0000882

H 1.8418072 2.3871450 -0.0001331

H 1.6480908 -1.9424700 0.0000901

O 3.2190415 0.2862867 -0.0000744

C 4.0311523 -0.9101617 -0.0002219

H 5.0586541 -0.5514932 -0.0006352

H 3.8360717 -1.4973503 0.9011689

H 3.8354383 -1.4976041 -0.9013065

C -2.3388291 0.4499473 0.0000544

H -2.7353426 1.4640479 0.0000065

C -3.2488239 -0.5813896 0.0001460

H -2.8957088 -1.6112983 0.0002024

C -4.7203089 -0.3960955 0.0001804

H -5.1634058 -0.8937352 -0.8748872

H -5.1633475 -0.8936040 0.8753525

H -5.0156563 0.6558232 0.0001123

**Table S3. Cartesian Coordinates of the Optimized Structure for the *trans*-β-Methylstyrene 14 Calculated at the RB3LYP/6-311G(2d,2p) level.**

Atom X Y Z

C -2.3446953 -1.0611250 -0.0000151

C -0.9673675 -1.2773516 -0.0000214

C -0.0580589 -0.2048735 0.0000000

C -0.5849355 1.1006876 0.0000459

C -1.9593553 1.3186802 0.0000445

C -2.8485515 0.2395842 0.0000155

H -3.0235710 -1.9100571 -0.0000339

H -0.5826036 -2.2949141 -0.0000531

H 0.0864038 1.9546386 0.0001009

H -2.3407346 2.3366735 0.0000774

H -3.9211850 0.4133614 0.0000241

C 1.3836673 -0.4984454 -0.0000165

H 1.6293717 -1.5616524 0.0001572

C 2.4007484 0.3760798 -0.0002433

H 2.1953477 1.4466169 -0.0005443

C 3.8511587 -0.0072708 0.0001022

H 4.3698212 0.3958574 0.8810799

H 3.9804908 -1.0947920 -0.0013487

H 4.3709972 0.3984744 -0.8789362

**Table S4. Cartesian Coordinates of the Optimized Structure for the Radical Cation of the *trans*-β-Methylstyrene 14^.+^ Calculated at the UB3LYP/6-311G(2d,2p) level.**

Atom X Y Z

C -2.3402405 -1.0675685 0.0000161

C -0.9829764 -1.3060347 -0.0000091

C -0.0482305 -0.2184674 -0.0000263

C -0.5573511 1.1237143 -0.0000116

C -1.9153623 1.3509796 0.0000078

C -2.8130501 0.2610381 0.0000236

H -3.0441295 -1.8932188 0.0000262

H -0.6028919 -2.3233893 -0.0000193

H 0.1255197 1.9658228 -0.0000290

H -2.3002103 2.3654089 0.0000088

H -3.8827626 0.4493562 0.0000399

C 1.3386800 -0.5319116 -0.0000363

H 1.5965108 -1.5897393 -0.0001125

C 2.3895295 0.3725806 0.0000020

H 2.1770241 1.4400517 -0.0001854

C 3.8154174 -0.0135430 0.0000043

H 4.3210588 0.4275584 0.8730894

H 3.9698918 -1.0946417 -0.0001430

H 4.3214929 0.4280668 -0.8724983

**Table S5. Cartesian Coordinates of the Optimized Structure for the Reaction Intermediate Figure 7-a Calculated at the UB3LYP/6-311G(2d,2p) level.**

Atom X Y Z

C 1.3766190 0.9820312 1.2349800

C 0.5543871 0.0196047 0.5708446

C 1.1887268 -0.8509457 -0.3635210

C 2.5434552 -0.7856038 -0.6105868

C 3.3360362 0.1672798 0.0770026

C 2.7266781 1.0530680 1.0051394

H 0.9186275 1.6595576 1.9502223

H 0.6008584 -1.5873216 -0.9017546

H 2.9934990 -1.4640829 -1.3252697

H 3.3572694 1.7712938 1.5181310

O 4.6482518 0.3130437 -0.0745303

C 5.3803916 -0.5313685 -0.9809222

H 6.4158726 -0.2040048 -0.9021025

H 5.2965444 -1.5802355 -0.6803882

H 5.0231542 -0.3950505 -2.0062917

C -0.8462640 -0.0251010 0.8678360

H -1.1724385 0.6489055 1.6539385

C -1.6636711 -1.1793331 0.6572948

H -1.2062431 -2.0357354 0.1706795

C -2.7425444 -1.5024368 1.6538343

H -2.2842006 -1.9139882 2.5650936

H -3.3076235 -0.6126784 1.9503171

H -3.4385647 -2.2547101 1.2737619

C -1.5018519 1.6382459 -0.5949043

H -1.0035247 2.5248066 -0.2128696

H -0.9282892 1.0610227 -1.3076781

C -2.8389068 -0.8007407 -1.2950304

H -1.8875980 -0.6911320 -1.8042493

H -3.3595097 -1.7344838 -1.4874700

C -2.8568206 1.5130718 -0.4991350

H -3.4217295 2.2712544 0.0398760

C -3.5503965 0.3091947 -0.8592781

C -5.0267785 0.2227249 -0.5846927

H -5.4051308 -0.7979910 -0.6788003

H -5.2781245 0.6043323 0.4123745

H -5.5732233 0.8477436 -1.3044461

**Table S6. Cartesian Coordinates of the Optimized Structure for the Reaction Intermediate Figure 7-b Calculated at the UB3LYP/6-311G(2d,2p) level.**

Atom X Y Z

C -1.2026981 -0.8684481 1.2271757

C -0.4995459 -0.0141860 0.3213492

C -1.2688735 0.6834656 -0.6539133

C -2.6410278 0.5571265 -0.7173821

C -3.3106409 -0.2882276 0.2006182

C -2.5670963 -1.0012272 1.1749180

H -0.6394364 -1.4148438 1.9788647

H -0.7753071 1.3364715 -1.3665561

H -3.1971952 1.1060753 -1.4675718

H -3.1063973 -1.6391861 1.8667674

O -4.6267339 -0.4810184 0.2309583

C -5.4844885 0.1951318 -0.7037599

H -6.4932505 -0.1285993 -0.4516657

H -5.4021637 1.2803487 -0.5876216

H -5.2453304 -0.0996323 -1.7304160

C 0.9132738 0.0983692 0.4310577

H 1.3475737 -0.4058511 1.2921199

C 1.7417565 1.2166041 -0.1507144

H 1.3846980 1.4265443 -1.1650785

C 1.5129886 2.5040757 0.6825329

H 0.4658044 2.8172844 0.6517239

H 1.7953995 2.3532737 1.7303888

H 2.1264443 3.3172999 0.2798955

C 1.6878439 -1.4591783 -1.0888731

H 1.0436752 -2.3339679 -1.1113398

H 1.4430357 -0.6919880 -1.8135023

C 3.2783399 0.9281213 -0.2190637

H 3.6763915 1.4537294 -1.1009423

H 3.7646189 1.4247975 0.6315631

C 2.9861898 -1.5682853 -0.5527857

H 3.3424556 -2.5629308 -0.2867788

C 3.7857647 -0.4912774 -0.2309395

C 5.1958924 -0.6932653 0.2344714

H 5.3191904 -0.3602773 1.2746157

H 5.5090165 -1.7377023 0.1634112

H 5.8885768 -0.0854924 -0.3636922

**Table S7. Cartesian Coordinates of the Optimized Structure for the Radical Cation of the Diels-Alder Adduct 3^.+^, Figure 7-c Calculated at the UB3LYP/6-311G(2d,2p) level.**

Atom X Y Z

C -1.2373062 -0.1954102 1.5040604

C -0.4757960 -0.1184160 0.3068822

C -1.1802914 -0.0737795 -0.9301131

C -2.5565651 -0.0720846 -0.9824024

C -3.2956846 -0.1360944 0.2314182

C -2.6112813 -0.1989005 1.4795978

H -0.7183526 -0.2427914 2.4568442

H -0.6214050 -0.0292644 -1.8598713

H -3.0663127 -0.0256041 -1.9373525

H -3.2040397 -0.2464245 2.3868903

O -4.6165155 -0.1478049 0.3098191

C -5.4393509 -0.0930510 -0.8751532

H -6.4639513 -0.1135539 -0.5084806

H -5.2523962 0.8352303 -1.4222983

H -5.2473001 -0.9637767 -1.5083117

C 1.0181574 -0.1227972 0.3576491

H 1.3183556 -0.1072479 1.4130490

C 1.7296588 1.0643368 -0.3547949

H 1.5677137 0.9470883 -1.4357129

C 1.1857091 2.4326251 0.0761008

H 0.1330540 2.5569645 -0.1988349

H 1.2709549 2.5679808 1.1613414

H 1.7502970 3.2392943 -0.4034874

C 1.5654567 -1.4600594 -0.2649758

H 1.1238476 -2.3318931 0.2329287

H 1.2406643 -1.5080743 -1.3134781

C 3.2440446 0.9776488 -0.0899143

H 3.7682818 1.6194497 -0.8134702

H 3.4782750 1.4211197 0.8920006

C 3.0567730 -1.5079471 -0.1693389

H 3.5161845 -2.4913167 -0.0878205

C 3.8443330 -0.4043496 -0.1265212

C 5.3400647 -0.4970445 -0.0614819

H 5.7268306 0.0385165 0.8164746

H 5.6871689 -1.5325423 -0.0165396

H 5.7967220 -0.0187757 -0.9385008

**Table S8. Cartesian Coordinates of the Optimized Structure for the Diels-Alder Adduct 3, Figure 7-d Calculated at the RB3LYP/6-311G(2d,2p) level.**

Atom X Y Z

C -1.2758012 0.1443594 1.5007628

C -0.4880066 -0.0734108 0.3596342

C -1.1591700 -0.2971332 -0.8479722

C -2.5542378 -0.3025643 -0.9297184

C -3.3162723 -0.0832936 0.2235220

C -2.6648256 0.1399717 1.4433313

H -0.7883519 0.3204515 2.4573635

H -0.5898493 -0.4707222 -1.7582885

H -3.0293104 -0.4784982 -1.8883668

H -3.2667689 0.3083092 2.3311438

O -4.6831729 -0.0672786 0.2646837

C -5.3910774 -0.2838177 -0.9438282

H -6.4503655 -0.2259910 -0.6859420

H -5.1588593 0.4851141 -1.6930959

H -5.1760628 -1.2738860 -1.3682970

C 1.0299968 -0.0830947 0.4492304

H 1.2980669 0.1533345 1.4900295

C 1.7268376 0.9801346 -0.4392257

H 1.5681582 0.6912333 -1.4896807

C 1.1646727 2.3927861 -0.2388600

H 0.1054760 2.4497570 -0.5065853

H 1.2613724 2.7068078 0.8088335

H 1.7102912 3.1191775 -0.8535431

C 1.5994127 -1.4861694 0.1366337

H 1.2703462 -2.1895217 0.9142828

H 1.1547404 -1.8644659 -0.7973520

C 3.2454472 0.9663193 -0.1783576

H 3.7495456 1.5517137 -0.9621426

H 3.4595797 1.5046870 0.7607901

C 3.1011717 -1.5069246 0.0280957

H 3.5794363 -2.4861763 0.0660628

C 3.8615612 -0.4135134 -0.1065807

C 5.3653826 -0.4785312 -0.1803997

H 5.8283648 0.1156509 0.6205195

H 5.7323217 -1.5063826 -0.0928686

H 5.7367030 -0.0630718 -1.1279387

**Table S9. Cartesian Coordinates of the Optimized Structure for the Reaction Intermediate Figure 7-e Calculated at the UB3LYP/6-311G(2d,2p) level.**

Atom X Y Z

C -3.2284563 0.6662771 -1.2180158

C -1.8919661 0.7783286 -0.8621041

C -1.3973619 0.1306416 0.3106004

C -2.3113582 -0.6392227 1.0939535

C -3.6479411 -0.7398616 0.7346117

C -4.1136952 -0.0892955 -0.4232387

H -3.5953461 1.1630376 -2.1088660

H -1.2252170 1.3637384 -1.4853775

H -1.9495268 -1.1364790 1.9886330

H -4.3333494 -1.3168991 1.3444660

H -5.1575764 -0.1691262 -0.7057113

C -0.0265491 0.2228339 0.7412901

H 0.1961411 -0.2554404 1.6901131

C 0.8581398 1.2964232 0.3554867

H 0.4800689 2.0229373 -0.3582506

C 0.7016404 -1.7449865 -0.2249144

H 0.1377919 -2.5103344 0.2988610

H 0.2171494 -1.3419038 -1.1042708

C 2.1863307 0.4641392 -1.3413616

H 1.2847276 0.2485100 -1.9041745

H 2.7541877 1.3208349 -1.6896133

C 1.8396352 1.8297589 1.3648323

H 1.3054803 2.4463249 2.1039362

H 2.3437357 1.0299646 1.9168544

H 2.5937671 2.4705088 0.9011640

C 2.0430377 -1.6013402 -0.0096407

H 2.5317039 -2.2164537 0.7421857

C 2.8125577 -0.5187618 -0.5751844

C 4.2573576 -0.3876515 -0.1785459

H 4.6873796 0.5664376 -0.4904716

H 4.3945213 -0.5017418 0.9042343

H 4.8461326 -1.1876111 -0.6503261

**Table S10. Cartesian Coordinates of the Optimized Structure for the Reaction Intermediate Figure 7-f Calculated at the UB3LYP/6-311G(2d,2p) level.**

Atom X Y Z

C -3.4017323 0.4552688 -0.9850435

C -2.0306749 0.6068087 -0.8626493

C -1.3274884 -0.0349131 0.1941013

C -2.0636782 -0.8426645 1.1055026

C -3.4355499 -0.9872047 0.9770190

C -4.1080305 -0.3399417 -0.0684082

H -3.9323185 0.9524161 -1.7909048

H -1.4964146 1.2230036 -1.5787348

H -1.5366381 -1.3401812 1.9153655

H -3.9876899 -1.5978541 1.6841891

H -5.1829323 -0.4544670 -0.1717915

C 0.0844320 0.1023546 0.3883524

H 0.4684500 -0.3625652 1.2946241

C 0.9312104 1.2095934 -0.1812488

H 0.6266532 1.3895578 -1.2176580

C 0.9435483 -1.4953291 -1.0418322

H 0.3079245 -2.3760650 -1.0721360

H 0.7264910 -0.7467821 -1.7942181

C 2.4701971 0.9358882 -0.1552516

H 2.9163231 1.4464089 -1.0230223

H 2.9052950 1.4524137 0.7112569

C 0.6400510 2.5153259 0.6062363

H -0.4071523 2.8128953 0.5079139

H 0.8670272 2.3956204 1.6712817

H 1.2654407 3.3244136 0.2141398

C 2.2065954 -1.5735607 -0.4316057

H 2.5527241 -2.5542412 -0.1068717

C 2.9889606 -0.4774947 -0.1111552

C 4.3787541 -0.6586291 0.4145578

H 4.4523328 -0.3104068 1.4546314

H 4.7069627 -1.6996744 0.3707319

H 5.0879523 -0.0475043 -0.1602470

**Table S11. Cartesian Coordinates of the Optimized Structure for the Radical Cation of the Diels-Alder Adduct 21^.+^, Figure 7-g Calculated at the UB3LYP/6-311G(2d,2p) level.**

Atom X Y Z

C -3.2908056 -0.0364587 -1.2501757

C -1.9126417 -0.0211151 -1.1301253

C -1.2986615 -0.1640759 0.1392630

C -2.1261742 -0.3700012 1.2738773

C -3.5074819 -0.3887044 1.1544060

C -4.0972790 -0.2236150 -0.1075530

H -3.7557483 0.0909542 -2.2227093

H -1.3003275 0.1170772 -2.0167338

H -1.6639472 -0.5005534 2.2484659

H -4.1300619 -0.5327214 2.0315824

H -5.1780268 -0.2489421 -0.2094627

C 0.1885436 -0.1314386 0.3029526

H 0.4133605 -0.1798800 1.3758860

C 0.9376056 1.0908645 -0.2911864

H 0.8424709 1.0442032 -1.3846916

C 0.8012557 -1.4351739 -0.3654416

H 0.3110127 -2.3475795 -0.0089748

H 0.5892313 -1.3670996 -1.4415075

C 2.4348581 0.9944822 0.0594469

H 3.0133719 1.6853310 -0.5719752

H 2.6027323 1.3666905 1.0853462

C 0.3630011 2.4296580 0.1915160

H -0.6731373 2.5581769 -0.1352138

H 0.3857263 2.4987647 1.2858686

H 0.9452629 3.2665201 -0.2085150

C 2.2615179 -1.4931984 -0.1209096

H 2.7172971 -2.4704678 0.0294505

C 3.0537350 -0.3708236 -0.0084889

C 4.5388633 -0.4726419 0.1097631

H 4.9009394 0.1007604 0.9742386

H 4.8834406 -1.5057355 0.1961607

H 5.0183857 -0.0220474 -0.7712817

**Table S12. Cartesian Coordinates of the Optimized Structure for the Diels-Alder Adduct 21, Figure 7-h Calculated at the RB3LYP/6-311G(2d,2p) level.**

Atom X Y Z

C -3.2560868 -0.3262961 -1.2737403

C -1.8731868 -0.2872589 -1.0970761

C -1.3093373 -0.1553335 0.1819838

C -2.1790227 -0.0659010 1.2765018

C -3.5644412 -0.1078518 1.1064346

C -4.1088825 -0.2380385 -0.1710625

H -3.6687633 -0.4270735 -2.2744849

H -1.2243629 -0.3583900 -1.9670676

H -1.7639827 0.0359491 2.2770660

H -4.2165910 -0.0380961 1.9734401

H -5.1865429 -0.2702888 -0.3078764

C 0.1977093 -0.1339203 0.3859516

H 0.3825693 0.0227644 1.4591777

C 0.9239073 1.0143817 -0.3616548

H 0.8483548 0.8094140 -1.4404325

C 0.8267727 -1.4941954 0.0048331

H 0.4643245 -2.2652196 0.6989980

H 0.4597923 -1.8059735 -0.9856559

C 2.4198337 1.0133809 0.0078133

H 2.9608873 1.6726405 -0.6875979

H 2.5516011 1.4772640 1.0002911

C 0.3077741 2.3923070 -0.0901978

H -0.7285235 2.4488821 -0.4362505

H 0.3139540 2.6190016 0.9840687

H 0.8783583 3.1795235 -0.5978700

C 2.3324053 -1.4710487 0.0039182

H 2.8344304 -2.4389828 -0.0028528

C 3.0685663 -0.3531869 0.0125712

C 4.5751463 -0.3775481 0.0405656

H 4.9649400 0.1628239 0.9151148

H 4.9643389 -1.4003062 0.0749306

H 4.9982688 0.1191252 -0.8440480

**2. General Information**

All solvents and reagents were from commercial source and were used without further purification. ^1^H-NMR spectra were collected on a 600 MHz NMR spectrometer using the deuterated solvent as an internal deuterium reference. Chemical shift data are given in δ units calibrated with residual protic solvent. The multiplicity of a signal is indicated as follows: s, singlet; d, doublet; t, triplet; q, quartet; m, multiplet. ^13^C-NMR spectra were collected on a 150 MHz spectrometer with proton decoupling using the deuterated solvent as an internal carbon reference. Chemical shift data are given in δ units calibrated with residual solvent. Only selected absorbances are reported in the IR spectra. HRMS analysis was performed in ESITOF or DARTTOF mode. The reaction yields were determined by NMR or GC-MS. Cyclic voltammograms were recorded at 2 mM of substrate using a glassy carbon working electrode, platinum counter electrode, and Ag/AgCl reference electrode in 1.0 M LiClO_4_/MeNO_2_ at 50 mV/s.

**3. Experimental and Spectra Information of Starting Materials**

A suspension of ethyltriphenylphosphonium iodide (5.19 g, 12.0 mmol) in THF (30 mL) was cooled to 0 °C under argon atmosphere, and then potassium *t*-butoxide (1.68 g, 15.0 mmol) was added. The reaction mixture was stirred for 30 min at 0 °C, then mesitaldehyde (1.47 mL, 10.0 mmol) was added and the solution was stirred for 6 h at room temperature. The reaction was quenched by the addition of saturated ammonium chloride aqueous solution (100 mL) and extracted with EtOAc (3 × 50 mL). The combined extracts were dried with Mg_2_SO_4_, filtered and concentrated in vacuo. The residue was purified by column chromatography (*n*-hexane–EtOAc, 20 : 1) affording the desired product as transparent oil (1.18 g, 7.33 mmol, 73%, *cis* : *trans* = 7 : 3). ^1^H-NMR (CDCl_3_, 600 MHz) δ 6.86 (1.4H, s), 6.85 (0.6H, s), 6.33-6.24(1H, m), 5.81 (0.7H, dq, *J* = 11.7, 6.9 Hz), 5.65 (0.3H, dq, *J* = 16.3, 6.9 Hz), 2.27 (2.1H, s), 2.26 (0.9H, s), 2.25 (2.1H, s), 2.16 (3.9H, s), 1.89 (0.9H, dd, *J* = 8.3, 2.1 Hz), 1.45 (2.1H, dd, *J* = 7.0, 2.1 Hz); ^13^C-NMR (CDCl_3_, 150 MHz) δ 136.0, 135.9, 135.8, 135.7, 134.8, 133.6, 130.0, 128.37, 128.35, 128.32, 127.8, 127.3, 21.0, 20.89, 20.87, 20.1, 18.8, 14.3; IR (NaCl, cm^-1^) 3007, 2965, 2918, 2857, 1614, 1443, 1378, 971; HRMS (DART-TOF) [M + H]^+^ calcd. for C_12_H_17_ 161.1330, found 161.1330.

A suspension of ethyltriphenylphosphonium iodide (1.51 g, 3.60 mmol) in THF (40 mL) and LiBr (624 mg, 3.60 mmol) was cooled to -78 °C under argon atmosphere, and then phenyllithium (1.8 M in diethylether, 6.48 mL, 3.60 mmol) was added. After stirring at room temperature for 0.5 h, the mixture was cooled to -78 °C again. A solution of *o*-anisaldehyde (362 μL, 3.00 mmol) in THF (10 mL) was added, subsequently phenyllithium (1.8 M in diethylether, 6.48 mL, 3.60 mmol) was added and the reaction mixture was stirred at -78 °C for 0.5 h. The reaction mixture was allowed to warm to room temperature, and then stirred for 0.5 h at room temperature. The mixture was cooled to -78 °C again and a solution of HCl (1.0 M in diethylether 3.60 mmol) was added. After 15 min potassium *t*-butoxide (448 mg, 4.00 mmol) was added to the solution, then the mixture was allowed to warm to room temperature and stirred for 1 h. The reaction was quenched by the addition of brine (100 mL) and extracted with EtOAc (3 × 50 mL). The combined extracts were dried over MgSO_4_, filtered and concentrated in vacuo. The residue was purified by column chromatography (*n*-hexane–EtOAc, 30 : 1) affording the desired product as transparent oil (262 mg, 1.77 mmol, 59%, *cis* : *trans* = 3 : 97). ^1^H-NMR (CDCl_3_, 600 MHz) δ 7.39 (1H, dd, *J* = 7.6, 1.8 Hz), 7.18 (1H, dt, *J* = 7.8, 1.8 Hz), 6.90 (1H, t, *J* = 7.3 Hz), 6.86 (1H, d, *J* = 8.3 Hz), 6.72 (0.97H, dd, *J* = 15.8, 1.8 Hz), 6.54 (0.03H, dd, *J* = 11.9 1.8 Hz), 6.22 (0.97H, dq, *J* = 15.8, 6.9 Hz), 5.85 (0.03H, dq, *J* = 11.7, 6.9 Hz), 3.84 (3H, s), 1.90 (2.91H, dd, *J* = 6.6, 1.8 Hz), 1.83 (0.09H, dd, *J* = 6.9, 1.8 Hz); ^13^C-NMR (CDCl_3_, 150 MHz) δ 156.2, 127.7, 127.1, 126.6, 126.5, 125.6, 120.7, 110.7, 55.4, 18.9; IR (NaCl, cm^-1^) 3034, 3003, 2961, 2938, 2833, 1598, 1489, 1033; HRMS (ESI-TOF) [M + Na]^+^ calcd. for C_10_H_12_ONa 171.0786, found 171.0787.

A suspension of ethyltriphenylphosphonium iodide (4.26 g, 11.5 mmol) in THF (50 mL) and HMPA (2.86 mL, 16.4 mmol) was cooled to 0 °C under argon atmosphere, and then KHMDS (0.5 M in toluene, 10.7 mL, 5.36 mmol) was added. After stirring at room temperature for 10 min, the mixture was cooled to -78 °C. *p*-anisaldehyde (1.00 mL, 8.22 mmol) was added dropwise, the reaction mixture was allowed to warm to room temperature, and then stirred for 1.5 h at room temperature. The reaction was quenched by the addition of saturated NH_4_Cl aqueous solution (100 mL) and extracted with EtOAc (3 × 50 mL). The combined extracts were dried over MgSO_4_, filtered and concentrated in vacuo. The residue was purified by column chromatography (*n*-hexane–EtOAc, 30 : 1) affording the desired product as transparent oil (612 mg, 4.13 mmol, 50%, *cis* : *trance* = 10 : 1). ^1^H-NMR (CDCl_3_, 600 MHz) δ 7.25 (2H, d, *J* = 8.9 Hz), 6.88 (1.8H, d, *J* = 8.9 Hz), 6.83 (0.2H, d, *J* = 8.3 Hz), 6.39-6.22 (1H, m), 6.09 (0.1H, dq, *J* = 15.8, 6.9 Hz), 5.70 (0.9H, dq, *J* = 11.7, 7.6 Hz), 3.82 (2.7H, s), 3.80 (0.3H, s), 1.89 (2.7H, dd, *J* = 7.2, 2.1 Hz), 1.86 (0.3H, dd, *J* = 6.2, 1.4 Hz); ^13^C-NMR (CDCl_3_, 150 MHz) δ 158.1, 130.3, 130.0, 129.2, 126.8, 125.1, 123.5, 113.9, 113.5, 55.2, 18.4, 14.6; IR (NaCl, cm^-1^) 3015, 2953, 2938, 2914, 2837, 1605, 1513, 1038; HRMS (ESI-TOF) [M + Na]^+^ calcd. for C_10_H_12_ONa 171.0786, found 171.0782.

A suspension of ethyltriphenylphosphonium iodide (3.89 g, 10.0 mmol) in THF (30 mL) was cooled to 0 °C under argon atmosphere, and then potassium *t*-butoxide (1.35 g, 12.0 mmol) was added. The reaction mixture was stirred for 30 min at 0 °C, then 2,4-dimethoxybenzaldehyde (1.02 mL, 6.0 mmol) was added and the solution was stirred for 6 h at room temperature. The reaction was quenched by the addition of saturated ammonium chloride aqueous solution (100 mL) and extracted with EtOAc (3 × 50 mL). The combined extracts were dried with Mg_2_SO_4_, filtered and concentrated in vacuo. The residue was purified by column chromatography (*n*-hexane–EtOAc, 5 : 1) affording the desired product as transparent oil (1.21 g, 6.76 mmol, 68%, *cis* : *trans* = 4 : 1). ^1^H-NMR (CDCl_3_, 600 MHz) δ 7.29 (0.2H, d, *J* = 8.9 Hz), 7.17 (0.8H, d, *J* = 7.6 Hz), 6.60 (0.2H, dd, *J* = 16.2, 2.0 Hz), 6.48-6.41 (2.8H, m), 6.09 (0.2H, dq, *J* = 15.8, 6.9 Hz), 5.76 (0.8H, dq, *J* = 11.3, 6.9 Hz), 3.81 (3H, s), 3.80 (2.4H, s), 3.79 (0.6H, s), 1.86 (0.6H, dd, *J* = 6.9, 1.4 Hz), 1.81 (2.4H, dd, *J* = 7.2, 1.4 Hz); ^13^C-NMR (CDCl_3_, 150 MHz) δ 159.8, 158.1, 157.2, 130.5, 127.1, 125.7, 125.2, 124.7, 124.5, 120.2, 119.1, 104.7, 103.7, 98.4, 98.3, 55.5, 55.4, 18.9, 14.6; IR (NaCl, cm^-1^) 3023, 3003, 2961, 2938, 2837, 1609, 1501, 1037; HRMS (ESI-TOF) [M + Na]^+^ calcd. for C_11_H_14_O_2_Na 201.0892, found 201.0894.

**4. Experimental and Spectra Information of Diels Alder Adducts**

**General procedure**

To a solution of lithium perchlorate (1.0 M) in MeNO_2_, styrene (80 mM, 1 equiv.) and diene (160 mM, 2 equiv.) were added. Two pieces of carbon felt were inserted into the solution and electrolysis was performed using an undivided cell with stirring at a constant potential of 1.0-2.0 V vs. Ag/AgCl under room temperature condition. Catalytic amount of electric charge was passed through the solution (the reactions can also be monitored by TLC), followed by dilution with EtOAc. The organic layer was washed with brine, dried over MgSO_4_, filtered and concentrated in vacuo. Purification was performed by silica gel column chromatography.

According to the General procedure, *trans*-anethole **1** (237 mg, 1.60 mmol), isoprene **2** (218. mg, 3.20 mmol), lithium perchlorate (2.13 g) and nitromethane (20 mL), were used and 0.1 F/mol of electricity was passed. Purification by silica gel chromatography (*n*-hexane–EtOAc, 30 : 1) afforded the Diels-Alder adduct (**3**) in product of transparent oil (3.33 g, 1.54 mmol, 96%; 98% determined by ^1^H-NMR). ^1^H NMR (CDCl_3_, 600 MHz) δ 7.08 (2H, d, *J* = 8.3 Hz), 6.84 (2H, d, *J* = 8.9 Hz), 5.44 (1H, br. s), 3.80 (3H, s), 2.29 (1H, td, *J* = 11.0, 5.5 Hz), 2.24-2.11 (2H, m), 2.08 (1H, dd, *J* = 17.2, 4.8 Hz), 1.93-1.84 (1H, m), 1.83-1.76 (1H, m), 1.69 (3H,s), 0.70 (3H, d, *J* = 6.2 Hz); ^13^C-NMR (CDCl_3_, 150 MHz) δ 157.8, 138.2, 133.8, 128.5, 120.9, 113.7, 55.2, 47.0, 39.8, 35.3, 34.0, 23.4, 20.2; IR (NaCl, cm^-1^) 3061, 3030, 2953, 2891, 2833, 1611, 1512, 1245; HRMS (ESI-TOF) [M + H]^+^ calcd. for C_15_H_21_O 217.1592, found 217.1590.

According to the general procedure, *trans*-anethole **1** (237 mg, 1.60 mmol), 1,3-butadiene (**4**) solution (0.64 M in cyclohexane, 5 mL, 3.20 mmol), lithium perchlorate (1.60 g) and nitromethane (15 ml) were used and 0.1 F/mol of electricity was passed. Purification by silica gel chromatography (*n*-hexane–EtOAc, 30 : 1) afforded the Diels-Alder adduct (**9**) as transparent oil (40% determined by ^1^H NMR). ^1^H-NMR (CDCl_3_, 600 MHz) δ 7.09 (2H, d, *J* = 8.7 Hz), 6.85 (2H, d, *J* = 8.7 Hz), 5.73 (2H, br. s), 3.80 (3H, s), 2.37 (1H, td, *J* = 10.5, 5.5 Hz), 2.28-2.12 (3H, m), 1.94-1.77 (2H, m), 0.70 (3H, d, *J* = 6.0 Hz); ^13^C-NMR (CDCl_3_, 150 MHz) δ 157.8, 138.2, 128.5, 126.9, 126.8, 113.7, 55.2, 47.0, 35.0, 34.9, 33.6, 20.2; IR (NaCl, cm^-1^) 3062, 3020, 2952, 2903, 2830, 1611, 1511, 1245; HRMS (ESI-TOF) [M + Na]^+^ calcd. for C_14_H_18_ONa 225.1255, found 225.1257.

According to the general procedure, *trans*-anethole **1** (237 mg, 1.60 mmol), 2,3-dimethyl-1,3-butadiene (**5**) (263 mg, 3.20 mmol), lithium perchlorate (2.13 g) and nitromethane (20 mL) were used and 0.1 F/mol of electricity was passed. Purification by silica gel chromatography (*n*-hexane–EtOAc, 30 : 1) afforded the Diels-Alder adduct (**10**) as transparent oil (96% determined by ^1^H-NMR). ^1^H-NMR (CDCl_3_, 600 MHz) δ 7.08 (2H, d, *J* = 8.9 Hz), 6.84 (2H, d, *J* = 8.9 Hz), 3.79 (3H, s), 2.33 (1H, td, *J* = 10.3, 5.5 Hz), 2.19-2.12 (1H, m), 2.12-2.04 (2H, m), 1.90-1.77 (2H, m), 1.64 (3H, s), 1.61 (3H, s), 0.69 (3H, d, *J* = 6.2 Hz); ^13^C-NMR (CDCl_3_, 150 MHz) δ 157.7,138.2, 128.4, 125.5, 125.3, 113.7, 55.2, 47.8, 41.8, 41.6, 34.2, 20.0, 18.7, 18.6; IR (NaCl, cm^-1^) 3065, 3030, 2953, 2911, 2830, 1614, 1512, 1245; HRMS (ESI-TOF) [M + H]^+^ calcd. for C_16_H_23_O 231.1749, found 231.1751.

According to the general procedure, *trans*-anethole **1** (237 mg, 1.60 mmol), *trans*-1,3-pentadiene (**6**) (218 mg, 3.20 mmol), lithium perchlorate (2.13 g) and nitromethane (20 mL) were used and 0.1 F/mol of electricity was passed. Purification by silica gel chromatography (*n*-hexane–EtOAc, 30 : 1) afforded the Diels-Alder adduct (**11**) as transparent oil (93% determined by ^1^H-NMR). ^1^H-NMR (CDCl_3_, 600 MHz) δ 7.06 (2H, d, *J* = 8.9 Hz), 6.84 (2H, d, *J* = 8.9 Hz), 5.75-5.72 (1H, m), 5.66-5.62 (1H, m), 3.80 (3H, s), 2.66 (1H, dd, *J* = 11.0, 5.5 Hz), 2.32-2.23 (2H, m), 2.13 (1H, sept, *J* = 5.5 Hz), 1.83-1.77 (1H, m), 0.83 (3H, d, *J* = 6.2 Hz), 0.74 (3H, d, *J* = 6.9 Hz); ^13^C NMR (CDCl_3_, 150 MHz) δ 157.6, 135.4, 133.5, 130.1, 125.1, 113.2, 55.2, 50.5, 35.5, 34.8, 26.5, 20.5, 16.8; IR (NaCl, cm^-1^) 3015, 2953, 2903, 2834, 1609, 1512, 1246, 825; HRMS (ESI-TOF) [M + Na]^+^ calcd. for C_15_H_20_ONa 239.1412, found 239.1413.

According to the general procedure, *trans*-anethole **1** (237 mg, 1.60 mmol), 2,4-dimethyl-1,3-pentadiene (**8**) (308 mg, 3.20 mmol), lithium perchlorate (2.13 g) and nitromethane (20 mL) were used and 0.1 F/mol of electricity was passed. Purification by silica gel chromatography (*n*-hexane–EtOAc, 30 : 1) afforded the Diels-Alder (**13**) adduct as transparent oil (73% determined by ^1^H-NMR). ^1^H-NMR (CDCl_3_, 600 MHz) δ 7.03 (2H, dd, *J* = 42.6, 6.9 Hz), 6.82 (2H, dd, *J* = 40.6, 6.9 Hz), 5.19 (1H, s), 3.80 (3H, s), 2.22-2.14 (2H, m), 2.09 (1H, dd, *J* = 17.9, 4.1 Hz), 1.75 (1H, dd, *J* = 18.2, 8.3 Hz), 1.66 (3H,s), 0.82 (3H, s), 0.77 (3H, s), 0.70 (3H, d, *J* = 5.5 Hz); ^13^C-NMR (CDCl_3_, 150 MHz) δ 157.7, 133.3, 130.3, 129.3, 113.2, 112.1, 57.4, 55.1, 40.5, 36.2, 30.0, 28.7, 24.7, 23.3, 20.8; IR (NaCl, cm^-1^) 3100, 3065, 3034, 2957, 2838, 1613, 1513, 1246; HRMS (ESI-TOF) [M + H]^+^ calcd. for C_17_H_25_O 245.1905, found 245.1904.

According to the general procedure, β-methyl-2,4,6-trimethylstyrene **15** (365 mg, 1.60 mmol), isoprene **2** (218 mg, 3.20 mmol), lithium perchlorate (2.13 g) and nitromethane (20 mL) were used and 0.1 F/mol of electricity was passed. Purification by silica gel chromatography (*n*-hexane–EtOAc, 30 : 1) afforded the Diels-Alder adduct **22** as transparent oil (*cis* : *trans* = 1 : 2, 13% determined by ^1^H-NMR). ^1^H-NMR (CDCl_3_, 600 MHz) δ 6.82 (1H, s), 6.78 (1H, s), 5.44 (1H, br. s), 3.00 (0.4H, td, *J* = 11.7, 5.5 Hz), 2.92 (0.6H, td, *J* = 11.7, 5.5 Hz), 2.45-2.21 (2H, m), 2.36 (1.2H, s), 2.35 (1.8H, s), 2.29 (3H, s), 2.24 (3H, s), 2.20-2.11 (0.4H, m), 2.05 (1.2H, dd, *J* = 16.7, 4.6 Hz), 1.96 (0.4H, dd, *J* = 17.6, 5.5 Hz), 1.84-1.73 (1H, m), 1.70 (1.8H, s), 1.66 (1.2H, s), 0.73 (1.8H, d, *J* = 6.4 Hz), 0.72 (1.2H, d, *J* = 6.4 Hz); ^13^C-NMR (CDCl_3_, 150 MHz) δ 137.9, 137.1, 137.0, 136.1, 134.8, 134.23, 134.16, 131.1, 131.0, 129.2, 121.22, 121.18, 43.6, 43.1, 40.7, 35.9, 35.6, 31.0, 30.8, 30.7, 23.6, 23.3, 21.9, 21.3, 20.6, 19.6, 19.5; IR (NaCl, cm^-1^) 3005, 2952, 2925, 2869, 2827, 1611, 914, 746; HRMS (DART-TOF) [M + H]^+^ calcd. for C_17_H_25_ 229.1956, found 229.1960.

According to the general procedure, β-methyl-2-methoxystyrene **16** (120 μL, 0.80 mmol), isoprene **2** (160 μL, 1.60 mmol), lithium perchlorate (1.06 g) and nitromethane (10 mL), 0.1 F/mol of electricity was passed. Purification by silica gel chromatography (*n*-hexane–EtOAc, 10 : 1) afforded the Diels-Alder adduct **23** as transparent oil (*cis* : *trans* = 1 : 10, 48% determined by ^1^H-NMR). ^1^H-NMR (CDCl_3_, 600 MHz) δ 7.18-7.12 (2H, m), 6.95-6.90 (1H, m), 6.86 (1H, d, *J* = 8.3 Hz), 5.45 (0.9H, br. s), 5.42 (0.1H, br. s), 3.81 (0.3H, s), 3.80 (2.7H, s), 2.99 (0.1H, dt, *J* = 10.3, 5.5 Hz), 2.91 (0.9H, q, *J* = 8.9 Hz), 2.16 (2H, br. s), 2.10-1.91 (2H, m), 1.87-1.78 (1H, m), 1.69 (2.7H, s), 1.67(0.3H, s), 0.73 (3H, d, *J* = 6.9 Hz); ^13^C-NMR (CDCl_3_, 150 MHz) δ 157.6, 157.5, 134.2, 133.9, 127.9, 126.6, 121.2, 120.8, 120.7, 110.7, 55.5, 39.9, 38.9, 35.1, 33.6, 32.8, 32.3, 23.6, 23.4, 19.9, 19.7; IR (NaCl, cm^-1^) 2953, 2922, 2880, 2833, 1501, 1493, 1242, 1034; HRMS (ESI-TOF) [M + Na]^+^ calcd. for C_15_H_20_ONa 239.1412, found 239.1412.

According to the general procedure, *trans*-β-methyl-3,4-dimethoxystyrene **18** (394 mg, 1.60 mmol), isoprene **2** (218 mg, 3.20 mmol), lithium perchlorate (426 mg) and nitromethane (20 mL) were used and 0.5 F/mol of electricity was passed. Purification by silica gel chromatography (*n*-hexane–EtOAc, 10 : 1) afforded the Diels-Alder adduct **24** as transparent oil (*cis* : *trans* = 1 : >10, 70% determined by ^1^H-NMR). ^1^H-NMR (CDCl_3_, 600 MHz) δ 6.80 (1H, d, *J* = 8.2 Hz), 6.73-6.68 (2H, m), 5.44 (1H, br. s), 3.87 (6H, s), 2.33-2.14 (3H, m), 2.09 (1H, dd, *J* = 17.4, 3.7 Hz), 1.94-1.75 (2H, m), 1.69 (3H, s), 0.72 (3H, d, *J* = 6.4 Hz); ^13^C-NMR (CDCl_3_, 150 MHz) δ 148.8, 147.1, 138.8, 133.8, 120.8, 119.6, 111.1, 110.7, 55.9, 55.8, 47.4, 39.8, 35.2, 34.0, 23.4, 20.2; IR (NaCl, cm^-1^) 2998, 2952, 2891, 2833, 1607, 1515, 1257, 746; HRMS (ESI-TOF) [M + Na]^+^ calcd. for C_16_H_22_O_2_Na 269.1518, found 269.1514.

According to the general procedure, *trans*-β-methyl-2,4-dimethoxystyrene **19** (394 mg, 1.60 mmol), isoprene **2** (218 mg, 3.20 mmol), lithium perchlorate (426 mg) and nitromethane (20 mL) were used and 0.5 F/mol of electricity was passed. Purification by silica gel chromatography (*n*-hexane–EtOAc, 10 : 1) afforded the Diels-Alder adduct (**25**) as transparent oil (*cis* : *trans* = 2 : 5, 21% determined by ^1^H-NMR). ^1^H-NMR (CDCl_3_, 600 MHz) δ 7.04-7.01 (1H, m), 6.48-6.43 (2H, m), 5.44 (0.9H, br. s), 5.41 (0.1H, br. s), 3.80 (3H, s), 3.78 (0.3H, s), 3.77 (2.7H, s), 2.88 (0.1H, td, *J* = 10.3, 5.5 Hz), 2.83-2.87 (0.9H, m), 2.14 (2H, s), 2.06 (1H, dd, *J* = 17.2, 4.8 Hz), 1.99 (0.9H, sept, *J* = 5.5 Hz), 1.90 (0.1H, sept, *J* = 5.5 Hz), 1.84-1.77 (1H, m), 1.69 (2.7H, s), 1.66 (0.3H, s), 0.73 (3H, d, *J* = 6.8 Hz); ^13^C-NMR (CDCl_3_, 150 MHz) δ 158.60, 158.47, 133.8,128.2, 126.5, 121.2, 120.7, 104.3, 98.5, 55.4, 55.3, 39.9, 39.4, 38.4, 35.0, 33.7, 32.9, 32.4, 23.5, 23.3, 19.9, 19.7; IR (NaCl, cm^-1^) 3000, 2953, 2918, 2833, 1613, 1505, 1257, 1041; HRMS (ESI-TOF) [M + Na]^+^ calcd. for C_16_H_22_O_2_Na 269.1518, found 269.1518.

**5. Theoretical Informatoin**

Geometry optimizations of all stationary points and frequency analyses were carried out at the RB3LYP or UB3LYP level of density functional theory with the 6-31G(d) basis set. The 6-311G(2d,2p) basis set was then adopted to calculate the single point energies of the obtained stationary points. No imaginary frequency was observed for *trans*-anethole **1**, the radical cation of trans-anethole **1^.+^**, *trans*-β-Methylstyrene **14**, the radical cation of *trans*-β-Methylstyrene **14^.+^**, the Diels-Alder adduct **3**, the Diels-Alder adduct **21**, the radical cation of the Diels-Alder adduct **3^.+^**, and the radical cation of the Diels-Alder adduct **21^.+^**.

**6. Copies of NMR spectra**
